# Effectiveness of Counseling Provided by Primary Care Doctors and Nurses in Increasing Glaucoma Screening Rates

**DOI:** 10.1155/2014/306795

**Published:** 2014-10-19

**Authors:** Witold Rezner, Anna Rezner, Sławomir Dutkiewicz

**Affiliations:** ^1^ZDROWIE Medical Center, Warszawska 34, 25-312 Kielce, Poland; ^2^Institute of Public Health, Faculty of Health Sciences, The Jan Kochanowski University in Kielce, Aleja IX Wieków Kielc 19, 25-317 Kielce, Poland

## Abstract

*Introduction.* An effective screening that can prevent glaucoma-related blindness largely depends on successful recruitment. This study was to assess the effectiveness of one-on-one counseling carried out by primary care doctors and nurses to increase glaucoma screening rates. *Material and Methods.* The study, carried out in an urban primary care center, involved 308 persons aged 35–87 years who were assigned to a doctor's, nurse's, or control group (*N* = 109, 110, and 89, resp.). Interventions by doctors and nurses included a brief one-on-one counseling session, while only a screening history was taken from controls. The number of people in each group with a positive screening status was assessed by telephone interview three months after the visit. *Results.* The percentage of persons in the nurse's counseling group who claimed being subjected to screening was more than four times higher than in the control group (20.9% versus 4.5%, *P* = 0.002). The doctor's interventions resulted in almost a tripled screening rate as compared to the control group (12.8% versus 4.5%, *P* = 0.052). There was no significant difference between screening rates in doctor's and nurse's groups (*P* = 0.212). *Conclusions.* In the studied population, counseling provided by nurses proved to be an efficacious method to encourage patients to undergo glaucoma screening.

## 1. Introduction

Glaucoma-related blindness affects about 8.5 million people worldwide [1]; however, it can be significantly reduced by early detection of glaucoma and prompt introduction of treatment, which may slow progression of the disease, especially the open-angle type [2, 3]. Glaucoma detectability and treatment results are still highly unsatisfactory not only in developing regions of the world but also in developed countries where glaucoma detection rate does not exceed 50% [4–7]. Population screening programs aimed at glaucoma risk groups may enable early detection of the disease and substantially reduce its negative personal and socioeconomic impact [8–11]. A key issue for screening is effective recruitment, which requires a wide range of strategies tailored to various characteristics of potential screening participants [12]. Although there is substantial data on the effectiveness of recruitment strategies for many screening programs worldwide, according to the authors' knowledge there is an absence of this kind of data concerning glaucoma screening. Primary care (PC), the most common form of health service in numerous countries, where treatments stay close to prevention, constitutes a natural environment for promoting prophylactic programs.

Our study was to assess the effectiveness of one-on-one counseling carried out by PC doctors and nurses to increase glaucoma screening rates measured three months after the intervention. Counseling was performed to encourage eligible patients to undergo free screening visit in specialist ophthalmology outpatient clinics independent from PC unit.

## 2. Materials and Methods

### 2.1. Glaucoma Screening Program in Poland

In Poland, a state-funded glaucoma screening program has been in place since 2006. This program, which was developed for participants aged 35 years and older who were neither diagnosed with glaucoma nor examined for glaucoma within the last 24 months, consists of a mandatory protocol performed by ophthalmologists. It includes an ophthalmological examination consisting of optic disc stereophotography of anterior and posterior segment structures, intraocular pressure assessment, and anterior chamber angle measurements.

### 2.2. Study Organization

Presented research belongs to the health and social survey group of studies related to human behaviours. During the conceptualization phase we assumed that the study plan comprising routine counseling procedures with no identified risk for patients performed by primary care doctors and nurses as a part of their everyday practice does not qualify it for formal ethical committee approval. Ethical issues, including respect to patients rights, were ensured by taking informed consent from every participant of the study and by adherence to routine primary care procedures to ensure privacy and other ethical aspects of patient management.

The study was conducted from March 2010 to May 2011 by primary care doctors and nurses according to a written protocol with detailed description of study procedures, including recruitment, aims of glaucoma screening encouragement, estimated duration of counseling sessions to encourage glaucoma screening, scope and form of information provided to patients, and procedures to assess the number of patients who underwent screening in specialist ophthalmology clinics after the counseling. A short training concerning the study protocol was given to the mentioned primary care staff. Every member of the study team was equipped with a written version of the protocol for referral. Every participant of the study was approached twice. First time during the recruitment phase which was followed by procedures assigned to intervention and control groups. Second approach occurred three months after initial participant's visit in the form of telephone interview to acquire information if the person was screened for glaucoma within the time from the visit.

### 2.3. Research Sample

Participants were recruited by primary care nurses from patients visiting a primary healthcare center within its standard everyday practice. Randomization was based on consecutive assignment of eligible participants to intervention and control groups. Eligibility for glaucoma screening was based on the above mentioned criteria for a state-founded glaucoma screening program.

To avoid randomization bias the study was not publicized, so the patients were generally unaware of it before their arrival to the center.

Patients who consented to the study and met the criteria for inclusion to at least one of four state-funded prophylactic programs (i.e., glaucoma, breast cancer, cervical cancer, or cardiovascular disease) were consecutively assigned to one of three groups (i.e., doctor's, nurse's, or control) according to their order of appearance at the health center. For a small portion of participants, due to organizational reasons, the group assignment included a few consecutively appearing patients into one particular group.

Considering patients who met inclusion criteria for at least one of the above mentioned prophylactic programs we established three databases for persons consecutively assigned to doctors, nurses, and control groups. For the purpose of this study, we chose from these groups only those patients, who qualified for glaucoma screening; therefore, in this paper, only the data related to participants eligible for glaucoma screening is presented and analyzed.

### 2.4. Research Methods and Tools

Study interventions were focused on providing patients with high quality information concerning medical and practical aspects of glaucoma screening. Duration of the counseling procedures performed by doctors and nurses was kept in accordance with instructions from the study protocol. Exact duration of every procedure was not measured, and adherence to the study protocol (including average duration of counseling procedures) was estimated on the base of interviews with doctors and nurses who participated in the study. We did not use standardized questions during counseling sessions. Questions as well as scope and form of information provided to patients were based on instructions contained in the study protocol.

Consultations performed by doctors required on average an additional 5 minutes after routine consultation and included taking a history of previous prophylactic programs, discussing the medical aspects of glaucoma screening along with its health benefits, informing on glaucoma screening providers, and answering questions.

The nurse consultations were also carried out in private rooms and were similar to the doctor's consultations, but they lasted on average 10 minutes and were not connected with consultation for other reasons.

Patients from both intervention groups were equipped with identical leaflets on glaucoma screening and with the list of contact details concerning local specialist ophthalmology outpatient clinics which were free glaucoma screening providers.

Patients assigned to the control group were asked by a nurse about their history of participation in prophylactic programs, though they were not advised on glaucoma screening.

In the doctor's group, the patients were consulted by one of three primary care doctors. One of the doctors interviewed the majority of patients, while the remaining two interviewed a dozen or so patients each. In the nurse's group, two nurse practitioners carried out the consultations, one of whom counseled twice as many patients as the other.

During operationalization phase we established that the efficacy of doctors and nurses interventions will be measured by means of patients declarations obtained by telephone interview. The interviews with all study participants were carried out three months after their first visit in the primary care center by the same primary care nurses who performed counseling procedures and included a standard question as follows: “Have you attended glaucoma screening examination during the last 3 months which constitute the period of time that passed from your visit 3 months ago in our primary care center?” Designatum of the efficacy of the interventions was the number of “yes” answers. Interview question was dichotomous and the question considered behaviours; therefore we assumed that the measurement is accurate.

All study participants were successfully contacted and interviewed.

### 2.5. Statistical Analysis

To assess the significance of differences between quantitative values, we used ANOVA for variables with normal distribution and for other variables we used Mann-Whitney* U* test (to compare two groups) and Kruskal-Wallis test (to compare three groups). Chi-square test was used to assess differences between qualitative variables. Significance level was *P* = 0.05.

MedCalc Statistical Software (version 12.7.7, MedCalc Software bvba, Ostend, Belgium) was used for statistical analyses.

## 3. Results

### 3.1. Demographic Characteristics

We enrolled 308 persons eligible for glaucoma screening who were mostly urban dwellers aged 35–87 years with average age of about 48, mostly with higher education and married. About three-quarters of study participants were women. The majority of participants had at least one child (Table 1).

### 3.2. Group Equivalence

There were no statistically significant differences in demographic characteristics between the compared study groups (Table 1).

### 3.3. The Effectiveness of Two Types of Interventions

The percentage of persons in the nurse's group who declared entering a glaucoma screening program within three months from intervention was more than four times higher than in the control group (Table 2) and in this intervention group it reached about one-fifth of persons who were eligible for glaucoma screening. Doctor's consultations resulted in an almost tripled increase in screening rate as compared to controls, but the difference did not reach statistical significance.

### 3.4. Socioeconomic Factors and Screening Adherence

Secondary analyses concerning relationship of socioeconomic factors with screening adherence did not reveal any statistically significant differences between persons who declared being up to date with screening and those who did not (Table 3).

## 4. Discussion

Presented data demonstrated the effectiveness of encouragement from PC nurses; however, we cannot unequivocally conclude on the effectiveness of doctor's consultation, although it was related to an almost three times higher declared screening rate than in controls. Lack of statistical significance of this apparent difference could have resulted from insufficient statistical power calculated on the assumption that the screening rate in the control group would be comparable to the very low screening rate observed in the general Polish population (oscillating around 3%), which was assessed using popular press data and personal observations in the inability to obtain information from other sources. Also, by taking the history of screening status in the control group, as well as by applying the procedure of an informed consent, we might have played some educational role that might have raised the screening rate above the expected level and contributed to the slightly underpowered constitution of the study. We must also remark that the effects of our interventions were measured as declared screening status, and this subjective type of measurement is a potential source of bias most probably producing somewhat exaggerated screening rates. As a potential source of bias, randomization procedure used in our study had some shortcomings due to organizational reasons described earlier; nevertheless it produced fairly equivalent study groups. Other potentially significant sources of bias within our study method were overall precision of the used measurement tools (questionnaires); problems with the assessment of reliability and validity of our results related to the lack of identified studies concerning the effectiveness of encouragement to participate in glaucoma screening in primary care setting, especially the assessment of external validity strongly associated with restrictions of sampling; the choice of professionals who performed interventions and conducted the study along with their communicativeness and engagement (the influence of professionals and investigators).

In our study, the percentage of individuals who declared positive screening status in intervention groups was substantially lower than that reported in another glaucoma epidemiology study in one of the largest Polish cities [13]. Authors reported that 6,000 invitations addressed to the target population resulted in 4,353 persons who entered the screening (80.8%); however, detailed description of recruitment procedures was not provided. This is the only paper with data on glaucoma screening recruitment in Poland that we have identified.

The one-on-one education strategy used in our study has also been demonstrated to be effective in numerous studies on recruitment for other screening schemes. The magnitude of increases in screening rates was comparable with our trial when controls were characterized with screening rates oscillating around 5% [14].

Intervention procedures in the study were designed with respect to conditions and limitations of PC environment so that interventions “passing effectiveness test” could be used in routine practice.

To discuss cost-effectiveness of nurses and doctors interventions we needed to consider labor costs and interventions efficacy measured as the number of patients who decided to undergo screening in specialist centers in comparison to controls. According to the data from the study setting, the cost of a 5-minute doctor counseling is comparable to the cost of a 10-minute counseling provided by a nurse. Consistent with our knowledge, this proportion may be common in other primary care centers in Poland. Assuming superior efficacy of nurses intervention versus doctors intervention as compared to controls and their comparable cost, one may put hypothesis that the cost-effectiveness of the first kind of intervention is higher; however, there is insufficient data from the study to reach a conclusion and further research is required in this field. To provide discussion in a wider context of cost-effectiveness of glaucoma management, including quality-adjusted life year cost analysis, more data would be required, especially concerning the effectiveness and cost of glaucoma screening procedures, as well as glaucoma treatment in specific settings; nevertheless, this study may provide useful input for further analyses.

## 5. Conclusions

In the studied population of urban primary care patients, one-on-one counseling provided by primary care nurses proved to be an efficacious method of encouragement to glaucoma screening. It seems to be also more cost-effective than that provided by doctors when the observed efficacy, time consumption, and labor costs of both types of interventions are considered.

## Figures and Tables

**Table 1:** Demographics of study participants.

Characteristics	Nurse's	Doctor's	Control	Chi-square; df	*P*
	consultation *n* = 110	consultation *n* = 109	group *n* = 89		
Sex					
Women	83 (75.5)	75 (68.8)	74 (83.1)	5.421; df = 2	0.066
Men	27 (24.5)	34 (31.2)	15 (16.8)		
Age					
Mean ± standard deviation	49.9 ± 12.2	46.2 ± 8.6	48.2 ± 10.0		
Range	35.0–87.0	35.0–68.0	35.0–70.0		0.153^a^
Median	48.5	45.0	46.0		
Education					
Elementary	5 (4.5)	1 (0.9)	3 (3.3)		
Secondary vocational	8 (7.2)	8 (7.3)	3 (3.3)	5.802; df = 6	0.446^b^
High school	33 (29.7)	35 (32.1)	35 (39.3)		
Higher	64 (58.2)	65 (59.6)	48 (53.9)		
Marital status					
Married	79 (72.1)	75 (68.8)	65 (73.0)	0.468; df = 2	0.791
Unmarried	31 (27.9)	34 (31.2)	24 (27.0)		
Children					
None	16 (14.6)	14 (12.8)	16 (18.0)	1.037; df = 2	0.595
One or more	94 (85.4)	95 (87.2)	73 (82.0)		
Place of residence					
Urban	96 (87.3)	104 (95.4)	78 (87.6)	5.103; df = 2	0.078
Rural	14 (12.7)	5 (4.6)	11 (12.4)		

^a^Kruskal-Wallis test.

^b^Three cells have expected value lower than 5; minimal expected group size is 2.60.

**Table 2:** The effectiveness of interventions as compared to controls.

	Nurse's consultation (*n* = 110)	Doctor's consultation (*n* = 109)	Control group (*n* = 89)	Statistical analysis	
				Chi-square; df	*P* ^a^
Patients who declared being up to date with glaucoma screening	23 (20.9)	14 (12.8)		1.559; df = 1	0.212
	23 (20.9)		4 (4.5)	9.947; df = 1	0.002
		14 (12.8)	4 (4.5)	3.761; df = 1	0.052

^a^With Yate's correction for continuity.

**Table 3:** Demographics of study participants according to their screening status.

Declared positive screening status	Nurse's consultation		Doctor's consultation		Control group	
	Yes (*N* = 23)	No (*N* = 87)	Yes (*N* = 14)	No (*N* = 95)	Yes (*N* = 4)	No (*N* = 85)

Sex						
Women	19 (82.6)	64 (73.6)	10 (71.4)	65 (68.4)	3 (75.0)	71 (83.5)
Men	4 (17.4)	23 (26.4)	4 (28.6)	30 (31.6)	1 (25.0)	14 (16.5)
Chi-square; df; *P* ^a^	0.389; 1; 0.533		0.000; 1; 1.000		0.000; 1; 1.000	
Age						
Mean ± standard dev.	53.3 ± 11.6	48.9 ± 12.2	50.1 ± 10.9	45.6 ± 8.1	47.3 ± 8.0	48.2 ± 10.2
Range	35–75	35–87	35–68	35–65	42–59	35–70
Median	53.0	45.0	49.0	44.0	43.5	46.0
95% CI	48.3; 58.4	46.3; 51.5	43.9; 56.4	44.0; 47.3	34.2; 59.8	46.1; 50.4
*F*-statistic; *P*	2.431; 0.122		3.407; 0.068		0.058; 0.810	
Mann-Whitney *U* test, *P*	0.065		0.160		0.890	
Education						
Elementary	3 (13)	2 (2.3)	— (0.0)	1 (1.0)	— (0.0)	3 (3.5)
Secondary vocational	2 (8.7)	6 (6.8)	1 (7.1)	7 (7.4)	1 (25.0)	2 (2.3)
High school	8 (34.9)	25 (28.4)	7 (50.0)	28 (29.5)	2 (50.0)	33 (38.8)
Higher	10 (43.5)	54 (62.1)	6 (42.9)	59 (62.1)	1 (25.0)	47 (55.3)
Chi-square; df; *P*	6.003; 3; 0.111^b^		2.507; 3; 0.474^c^		6.725; 3; 0.081^d^	
Marital status						
Married	18 (78.3)	61 (70.1)	8 (57.1)	67 (70.5)	2 (50.0)	63 (75.3)
Unmarried	5 (21.7)	26 (29.9)	6 (42.9)	28 (29.5)	2 (50.0)	22 (24.7)
Chi-square; df; *P* ^a^	0.262; 1; 0.609		0.490; 1; 0.484^e^		0.236; 1; 0.627^f^	
Children						
None	1 (4.3)	15 (17.2)	1 (7.1)	13 (13.7)	— (0.0)	16 (18.8)
One or more	22 (95.6)	72 (82.8)	13 (92.9)	82 (86.3)	4 (100.0)	69 (81.2)
Chi-square; df; *P* ^a^	1.506; 1; 0.220^g^		0.065; 1; 0.799^h^		0.085; 1; 0.770^i^	
Place of residence						
Urban	18 (78.3)	78 (89.6)	14 (100.0)	90 (94.7)	4 (100.0)	74 (87.1)
Rural	5 (21.7)	9 (10.3)	— (0.0)	5 (5.3)	— (0.0)	11 (12.9)
Chi-square; df; *P* ^a^	1.224; 1; 0.269^j^		0.038; 1; 0.846^k^		0.000; 1; 1.000^l^	

^a^With Yate's correction for continuity.

^b^Three cells have expected value lower than 5; minimal expected group size is 1.05.

^c^Four cells have expected value lower than 5; minimal expected group size is 0.13.

^d^Six cells have expected value lower than 5; minimal expected group size is 0.13.

^e^One cell has expected value lower than 5; minimal expected group size is 4.37.

^f^One cell has expected value lower than 5; minimal expected group size is 11.85.

^g^One cell has expected value lower than 5; minimal expected group size is 3.35.

^h^One cell has expected value lower than 5; minimal expected group size is 1.80.

^i^Two cells have expected value lower than 5; minimal expected group size is 0.72.

^j^One cell has expected value lower than 5; minimal expected group size is 2.93.

^k^Two cells have expected value lower than 5; minimal expected group size is 64.

^l^Two cells have expected value lower than 5; minimal expected group size is 0.49.

## Abbreviations

PC: Primary care.
